# Thickness profiling of electron transparent aluminium alloy foil using convergent beam electron diffraction

**DOI:** 10.1111/jmi.13137

**Published:** 2022-08-22

**Authors:** Artenis Bendo, Masoud Moshtaghi, Matthew Smith, Zelong Jin, Yida Xiong, Kenji Matsuda, Xiaorong Zhou

**Affiliations:** ^1^ School of Materials University of Manchester Manchester UK; ^2^ Department of General and Analytical Chemistry Montanuniversität Leoben Leoben Austria; ^3^ Graduate School of Science and Engineering University of Toyama Toyama Japan

**Keywords:** aluminium alloy, convergent beam electron diffraction (CEBD), extinction distance, Kossel–Möllenstedt (K–M) fringe, thickness fringe, transmission electron microscopy (TEM), two‐beam condition

## Abstract

Convergent beam electron diffraction (CBED) was used to profile the thickness of aluminium alloys foils prepared by using the twinjet electropolishing method. The two‐beam CBED condition was obtained by exciting the {200} and {111} aluminium diffracted g‐vector. The aluminium alloy foil thicknesses were calculated at different distances from the sample hole edge. In areas where only one Kossel–Möllenstedt (K–M) minima fringe was obtained, the thickness was determined by matching the experimental with simulated convergent beam diffraction patterns. In areas far away from the sample edge, the thickness of foils was high enough to generate at least two (K–M) minima fringes, required for linear regression fitting.

## Lay description

To achieve highest possible magnification with the highest resolution and observe the nanometer‐scale features of the microstructure in aluminum alloys, it is important to obtain samples which are as thin as we possibly can get to. The samples must be so thin that they can be transparent to the electron beam. To have a clear picture of how profile of a thin sample should look like, advanced microscopes were used where the electrons are concentrated in very narrow areas over the sample. The electron beam going through the sample, experiences a physic phenomenon known as diffraction, which is recorded below the sample, and thickness is calculated out of it. By concentrating the electron beam to different points over the sample, thickness profile was reconstructed. This is imperative for building up reference profiles for check and compare of samples used in transmission electron microscopy.

## 1 INTRODUCTION

Converging electron beam to areas around 1 nm or less in diameter makes the dynamical effects to become significant. Dynamical effects give rise to K–M fringes, which appear inside the electron‐diffracted spots.^1^ Intensity oscillations inside the diffracted spots follow the equation^2^:

(1) $$\;I_{g}=\;\frac{1}{\left(1+{\;\xi }_{g}^{2}\;s^{2}\right)}\;\mathrm{si}n^{2}\left(\pi t\;\sqrt{\frac{1}{\xi_{g}^{2}}+\;s^{2}}\right),$$

where *s* is the excitation error/deviation parameter from the perfect Bragg condition, $\xi_{g}$ is the extinction distance and *t* is the sample thickness. In case the sample is tilted to the exact Bragg angle of diffraction, the deviation parameter from the Bragg condition, s, becomes zero. If *s* = 0, Equation (1) simplifies to:

(2) $$\;I_{g}=\;\mathrm{si}n^{2}\left(\frac{\pi \;t}{\xi_{g}}\right),$$

According to Equation (2), the intensity $I_{g}$ at *s* = 0 is equal to zero when the thickness *t* is equal to an integer number *n* of the extinction distance $\xi_{g}$.

(3) $$t\;=\;n\;\xi_{g}.$$

Every time the sample thickness equals the extinction distance, one pair of minima K–M fringes will appear in the intensity oscillations in the CBED disks. When the electron beam reaches one extinction distance depth, the amplitude of the incident beam becomes zero, and that of the diffracted beam is maximal and vice versa. The above equation is rearranged taking into consideration the effective extinction distance is different from the extinction distance because it considers the case when the exact Bragg angle of diffraction is not satisfied. The effective extinction distance is smaller than the reference one and it makes the fringes to appear in smaller crystal thicknesses. In that case, Equation (3) is rearranged to^3^:

(4) $$\left(s_{i}^{2}+\frac{1}{\xi_{g}^{2}}\right)\;\;t^{2}=\;n_{i}^{2},$$

where $s_{i}$ is the deviation parameter for each *i*th minima fringe from the exact Bragg condition, which is the position of the fringe at the centre of the convergent diffraction disks for which the deviation parameter *s* = 0, and *n_i_* is an integer number which we arbitrarily assign to each fringe. By dividing with $n_{i}^{2}$ on both sides and rearranging the Equation (4), the following one is obtained^4^, ^5^, ^6^, ^7^, ^8^, ^9^, ^10^, ^11^, ^12^, ^13^:

(5) $$\;{\left(\frac{s_{i}}{n_{i}}\right)}^{2}=\;-{\left(\frac{1}{\xi_{g}}\right)}^{2}{\left(\frac{1}{n_{i}}\right)}^{2}+\;{\left(\frac{1}{t}\right)}^{2}.$$

Equation (5) is the equation that has practical use for measuring the crystal thickness. Plotting the (*s_i_*/*n_i_*)^2^ with respect to (1/*n_i_*)^2^, the slope equals the –(1/*ξ* ***_g_***)^2^ term and the interception equals the (1/*t*)^2^ term.^14^ The slope and the interception are used to calculate the extinction distance $\xi_{g}$ and thickness *t*, respectively.

One main use of CBED is for measuring crystal thicknesses. Atomic‐scale resolution imaging and analytical TEM analysis of the microstructure depend directly on the thickness of TEM sample. TEM samples must have wide areas that are electron‐beam transparent with regions next to the hole edges being thin enough for carrying out atomic‐scale investigation. Reports on the quality of the aluminium TEM samples with respect to the twinjet electropolishing parameters have not yet provided a reference picture of what the TEM aluminium sample thickness profile looks like.^15^, ^16^ In this work, the thickness profiling of TEM aluminium alloy samples prepared with the common twinjet electropolishing were carried out. The TEM samples were prepared using the same acidic solution and electropolishing parameters. Sample thicknesses were measured by means of two‐beam CBED method at different distances from the twinjet hole edge. The obtained thickness profile in this work can serve as a reference point for match and compare with TEM samples prepared using the same method.

## 2 EXPERIMENTAL PROCEDURE

The first alloy (alloy 1) based on Al‐Zn‐Mg system was solution treated at 475°C for 1 h and subsequently water quenched. The alloy was then aged to near peak‐strength condition at 120°C after initially 4 days natural aging. The second alloy (alloy 2) based on Al‐Mg‐Si system was cast as a billet with diameter of 153 mm. The chemical compositions of the alloys were determined using spark emission spectroscopy (SES) and it is shown in the Table 1 in the supplementary material. The microstructure of the investigated alloys is shown in the Figure 1 in the supplementary material.

Alloy disks were punched out from around 70 μm thick mechanical polished foils which were then electropolished in a twinjet electropolishing machine, using a solution of 1/3 nitric acid (HNO_3_) and 2/3 methanol (CH_3_OH) at a temperature range between −20 and −30 C, 17 V and polishing time 2 min.

The thickness measurements were carried out using two transmission electron microscopies, in a Topcon EM‐002B equipped with LaB_6_ thermal electron gun operated at 120 kV and in a FEI Tecnai TF30 equipped with Field Emission Gun (FEG) operated at 300 kV. Double‐tilt specimen holders were used in both TEM for tilting the TEM sample into two‐beam CBED condition. The electron beam was converged to a fine spot over an area brought to eucentric height and focused to minimal contrast. The condenser aperture size used in both TEM was 100 μm. CBED simulations were carried out in MacTempas^TM^ simulation software using half‐convergence angle of 1.77 mrad and the Scherzer defocus of –50 nm. The convergence angle was measured experimentally on the obtained TEM results with respect to the Au‐Pd nanocrystal reference TEM sample.

## 3 RESULTS

Thickness measurements are carried out on TEM samples prepared from two different aluminium alloy compositions. The main results are shown here and the extra ones for comparison in the supplementary material. TEM foil thicknesses are measured using two‐beam CBED at different distances from the hole edge. All CBED patterns are obtained by either exciting either {200} or {111} aluminium diffracted spots in the two‐beam condition.

**FIGURE 1:**
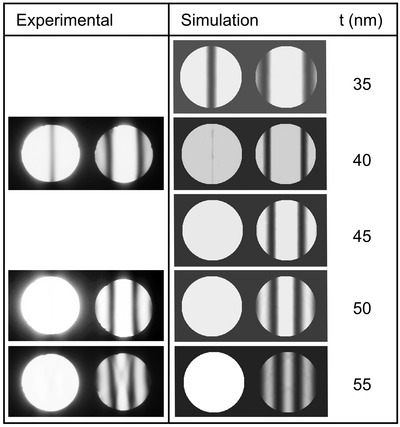
Experimental and simulated two‐beam CBED patterns for the exited {200} Al spot, 120 keV acceleration voltage and 1.77 mrad half‐convergence angle for a range of thicknesses along the 501 Al zone axis

Figure 1 shows the experimental and simulated two‐beam CBED patterns obtained by exciting the {200} Al spot in the areas near the twinjet hole edge for alloy 1. The TEM foil is thin enough as only one pair of K–M minima fringes appear inside the CBED disks. The thickness is found by matching the simulations with the experimental results. Simulations are carried out along the 501 zone axis for pure aluminium at room temperature for thickness ranging from 35 to 55 nm. The diffractions show that the deviation from the exact Bragg angle of the first minima fringe decreases as the sample thickness increases. This indicates that the frequency of intensity oscillation of the rocking curve increases with increase in sample thickness.

**FIGURE 2:**
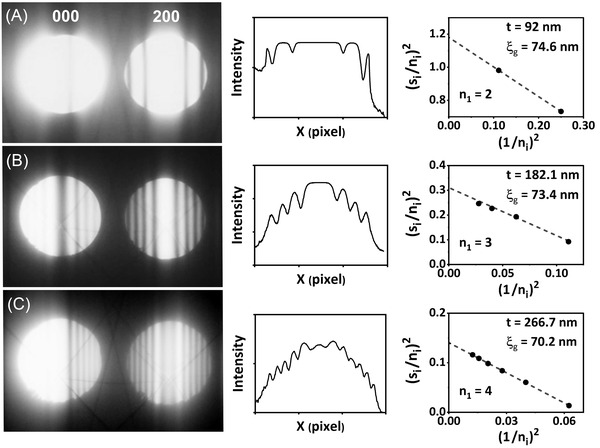
Two‐beam CBED patterns for the {200} Al exited spots, the intensity profile/rocking curve oscillations of the {200} diffracted spots and the linear regression fitting plots used for measuring the TEM foil thickness corresponding to measured thicknesses of (A) 92 nm, (B) 182 nm and (C) 266 nm

Increase in thickness will increase the number of K–M minima/maxima fringes generated inside the CBED disks. When more than two K–M minima fringes appear inside the CBED disks, the linear regression fitting is feasible for calculating the sample thickness. Figure 2 shows an example of the calculations of the two‐beam CBED patterns with {200} Al excited spot. The sample thickness in the CBED probing points at distances further away than 10 μm from the hole edge for alloy 1 and is large enough that at least two minima/maxima extinction fringes are generated. This is required for the linear regression fitting.^4^ The deviation parameter of the minima/maxima is measured using the following equation^4^:

(6) $$\;s_{i}=\;\left(\frac{\lambda }{d^{2}}\right)\;\left(\frac{\Delta \theta_{i}}{2\theta_{d}}\right),$$

where *λ* corresponds to the wavelength of the electron beam which is 0.00352 nm for 120 kV and *d* is the interplanar spacing of the {200} Al planes which is 0.2025 nm. The exact angle of the Bragg diffraction, $2\theta_{d}$, is the distance between the {000} and {002} Al spots and the $\Delta \theta_{i}$ is the distance from the exact angle of Bragg diffraction to each minimum/maxima K–M fringe. The ratio of angles $\Delta \theta_{i}$ over $2\theta_{d}$ is the ratio of distances measured directly on the CBED patterns and therefore there is no need for calibration of the camera length or the magnification since Equation (6) is the ratio of the same units.^14^ The deviation from the exact Bragg condition, *s_i_*, is measured for every minimum fringe from the intensity‐pixel oscillation plots seen in the Figure 2, and all the obtained values are inserted in the Equation (5). The corresponding (*s_i_*/*n_i_*)^2^ versus (1/*n_i_*)^2^ plots for each CBED patterns are generated as seen in the examples given in the Figure 2. The dashed lines are the linear fitting lines done over the data points. The foil thickness, *t*, is calculated from the interception of the linear fitted line with the (*s_i_*/*n_i_*)^2^ axis and the extinction distance $\xi_{g}$ is extracted from the slope.^6^, ^14^ The number of data points in the (*s_i_*/*n_i_*)^2^ versus (1/*n_i_*)^2^ plot corresponds to the number of minimum fringes in the two‐beam CBED patterns. Increase in thickness will increase the number of minima fringes which will increase the number of data points in the plot, which in turn will increase the accuracy of the linear fitting. The difference between the calculated extinction distance and the reference one, ξ_200_ = 73.7 nm, should be minimal. The difference stems from a range of factors, which are introduced in the discussion part. A more detailed description of the experimental procedure is given in the reference.^14^

**FIGURE 3:**
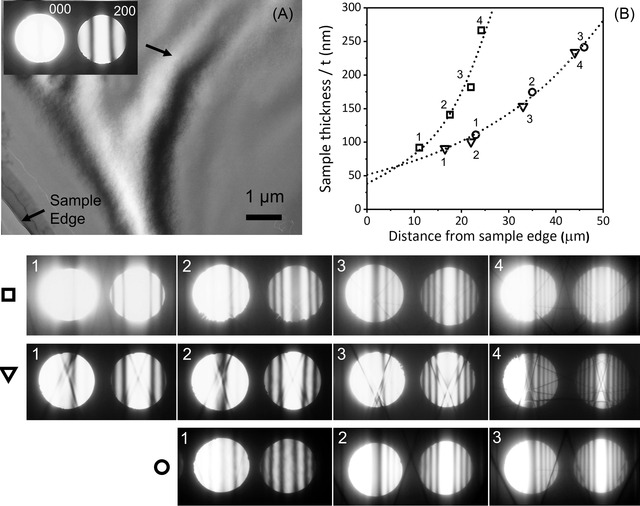
Thickness measurement in alloy 1. (A) Bright field TEM image, defocused on purpose to enhance the bend contours. (B) The TEM foil thickness as a function of the distance from the sample edge. The CBED patterns with the excited {200} Al spot corresponding to three probing paths indicated with Δ, □ and ○

Figure 3 shows all the CBED patterns for three sets of sample pathing in alloy 1. Figure 3A shows a bright field TEM image slightly defocused to enhance the contours and the sample edge and the probing point where the CBED pattern is obtained is indicated. Figure 3B shows the sample thickness as a function of the distance from the hole edge for alloy 1. The increase in sample thickness is shown to be exponential in all group of measurements. The extrapolation of the exponential fitting down to the hole edge gave a thickness range of 40–50 nm which fits perfectly well with the experimental CBED patterns, and the simulations matching shown in the Figure 3. Indicated with square, triangle and circle are all the CBED patterns acquired at distance more than 10 μm away from the hole edge in the TEM foils. The exponential increase in thickness of the TEM foils was validated to be a reasonable approximation for the TEM samples feasible for atomic‐scale imaging which was not the case for the contrary as shown in the Figure 2 in the supplementary material.

## 4 DISCUSSION

The foil thickness measurement using CBED method is assumed to be within ±2% accuracy.^4^ The extinction distances measured in all CBED measurements deviated slightly from the reference ones, as shown in the insets of the (*s_i_*/*n_i_*)^2^ versus (1/*n_i_*)^2^ plots in the Figure 2. The source of error may be due to a range of factors:

(a) Measurement error. The minima are not located precisely at the mid distance, but it is slightly displaced, especially in the first fringe. However, intensity line profiling can reduce the bias in measurement by increasing the accuracy of locating it.^9^^,^ 11 As the effect of other factors highlighted below is increased, the error due to measurement increases as well.
(b) Reference extinction distance. The extinction distance $\xi_{g}$ depends on the volume of the unit cell *V*, the Bragg angle of scattering $\theta_{B\;}$, the wavelength of the electron beam λ and the structure factor of the unit cell $F_{g}$ ^14^:
(7) $$\;\xi_{g}=\;\frac{\pi \;V\;\cos\theta_{B}}{\lambda \;\left|F_{g}\right|}.$$

In this work it is assumed that the TEM foil was pure Al, since the alloy was aged long enough for the solute elements to be incorporated in the metastable phases and leaving enough space for the convergent beam to probe between the precipitates where the solute concentration is dilute. However, it is reported that in addition to the oxidation layer, which forms on the surface of the TEM samples, there might be solute enriched layers which segregate between the oxide layer and the matrix.^17^ This will alter the structure factor and the volume of the unit cell described in the Equation (7), which in turn will influence the extinctions distance and the absorbing coefficient.

(c) Debye–Waller/temperature factor. The long exposure of the specimen by the electron beam causes increase in temperature. Increased thermal vibrations will increase the extinction distance. At ambient temperature it is claimed that the thermal vibration effect would not raise the $\xi_{g}$ by more than 5%.^5^ The $\xi_{g}$ due to thermal vibrations increases for higher order reflections since the vibration amplitude of the atoms relative to the interplanar spacing increases as the interplanar spacing decreases. For low‐order reflections which correspond to high interplanar spacings, the thermal vibration effect in the $\xi_{g}$ is small.^5^
(d) Absorption. Absorption causes a decrease in contrast in the CBED patterns contrast.^5^, ^12^ The absorption shifts the maxima in higher $\Delta \theta_{i}$ values and the minima in lower $\Delta \theta_{i}$ values. The biggest effect is for the first minima/maxima.^7^, ^8^ Therefore, the biggest error comes from the measurement of the position of the fringe with the lowest $\Delta \theta_{i}$, which might be significantly displaced.^12^ The absorption effect goes to nearly zero for the fringes located at highest $\Delta \theta_{i}$. Equation (5) which is used practically for thickness calculation does not consider the absorption effect.^8^ Ideally, as Equation (5) states, the intensity of the minima fringes must be zero since the absorption is zero.^9^ However, the experimental intensity profiles showed intensity to be non‐zero in the minima fringes as seen illustrated in the rocking curves in the Figure 3. The non‐zero intensity of minima is reported to be due to plasmon scattering.^18^ Overall, the effect of absorption increases the intensity of minima from zero to some value, and at the same time shifts the minima to larger $\Delta \theta_{i}$.^8^ Absorption effect is significant in low‐order reflections.
(e) Multibeam scattering. As the thickness increases the contrast is reduced because the amount of inelastically scattered electrons increases.^12^ Multibeam scattering reduces in high‐order diffracted beams, and it is intense for low g‐vectors because the scattering power is high for low angles of diffractions.^1^, ^4^, ^5^, ^6^, ^7^

In this work, the {200} and {111} Al reflections were exited for the two‐beam CBED diffractions which are low‐order reflections or short diffracting g‐vectors. Being a low‐order reflection, the thermal vibration effect is small, meanwhile the absorption and the multibeam scattering effects are high.^4^, ^5^ Therefore, errors would mainly stem from absorption and the multibeam scattering effects. Neither absorption, nor multibeam scattering is included in Equation (5) which was used for the thickness measurements.^7^, ^10^ Another source of error is the reference extinction distance. Though the extinction distances are available for some pure metals, for alloys it is generally not known and in this work the extinction distance of pure Al was used as a reference.^10^ However, taking into consideration all the errors, it is still reasonable to assume that the measurement errors in thickness is negligible compared to the huge differences between the TEM sample thickness profiles as seen by directly comparing Figure 3B and Figure 2 in the supplementary material.

A large number of thickness profiling of TEM foils based on the two‐beam CBED were carried out in samples from two different aluminium alloy compositions. It was revealed that high‐quality TEM samples had a thickness of around 40–50 nm right next to the twinjet hole edge, with areas in thickness of up to 100 nm extending tens of micrometres away from the twin jet hole edge and thickness increasing exponentially as a function of distance form sample edge. In contrast, other TEM samples started with a thickness of around 200 nm right to the edge of the sample hole and increased abruptly parabolically to several hundred nanometres within a few micrometre range from the sample edge as seen in the Figure 2 in the supplementary material.

## 5 CONCLUSIONS

Thickness profiling was successfully carried out using two‐beam CBED diffraction method by taking advantage of measuring the crystal thickness precisely at the diffraction point. Measurement based on linear fitting on the CBED patterns with more than two minima K–M fringes and simulations match for CBED patterns with one minimum K–M fringe revealed that the thickness of aluminium alloy foils prepared by twinjet electropolishing which are suitable for atomic‐scale resolution started with 40–50 nm thickness next to the hole edge and exponentially increases over tens of micrometre range.

## Supporting information

Figure S1

Figure S2

Supplementary Materials
