# Supplementary figures and images for: Thickness profiling of electron transparent aluminium alloy foil using convergent beam electron diffraction
Source: J Microsc. 2022 Aug 22;288(1):10–5. doi: 10.1111/jmi.13137 (PMC9804362; doi:10.1111/jmi.13137)

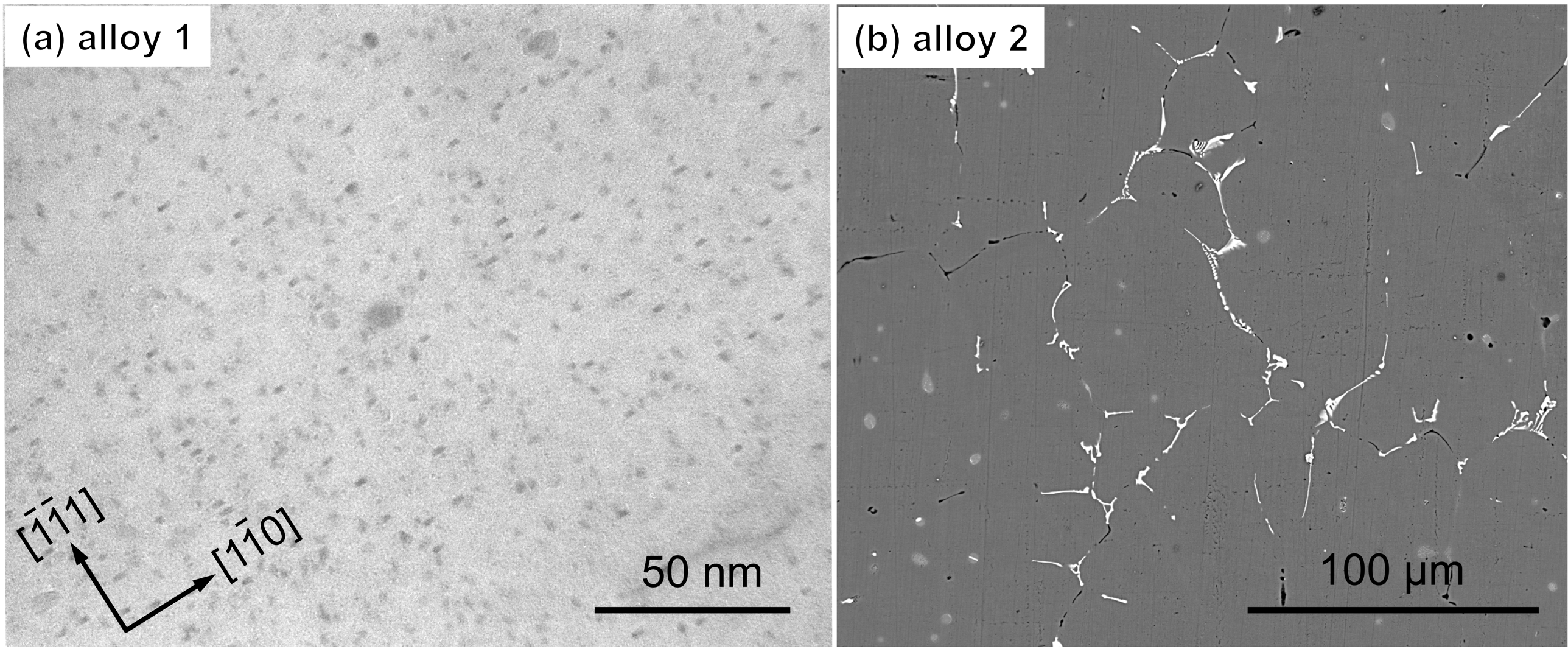

Supplement: Supplementary file 1 — Figure S1 [file JMI-288-10-s002.tif]
