# Supplementary material for: Thickness profiling of electron transparent aluminium alloy foil using convergent beam electron diffraction
Source: J Microsc. 2022 Aug 22;288(1):10–5. doi: 10.1111/jmi.13137 (PMC9804362; doi:10.1111/jmi.13137)
Supplement: Supplementary file 3 — Supplementary Materials [file JMI-288-10-s003.docx]

**Supplementary Materials**

**alloy 1**

Al - 1.8 Zn - 1.0 Mg (at. %)

**alloy 2**

|  | Al | Si | Mg | Mn | Fe | Cu | Cr | Ti | B | V | Hf | Ni, Ga, Zn, Pb, Sr, Y |
| --- | --- | --- | --- | --- | --- | --- | --- | --- | --- | --- | --- | --- |
| wt.% | **97.4** | **1.02** | **0.684** | **0.478** | **0.181** | 0.074 | 0.067 | 0.036 | 0.002 | 0.011 | 0.018 | 0.015 |
| SD | **0.018** | **0.003** | **0.007** | **0.003** | **0.006** | - | - | 0.001 | - | - | 0.001 | 0.002 |

Table 1. Compositional analysis of the A6082 alloy using Spark Emission Spectroscopy (SES). The results are reported in wt.% and the corresponding standard deviations (SD).


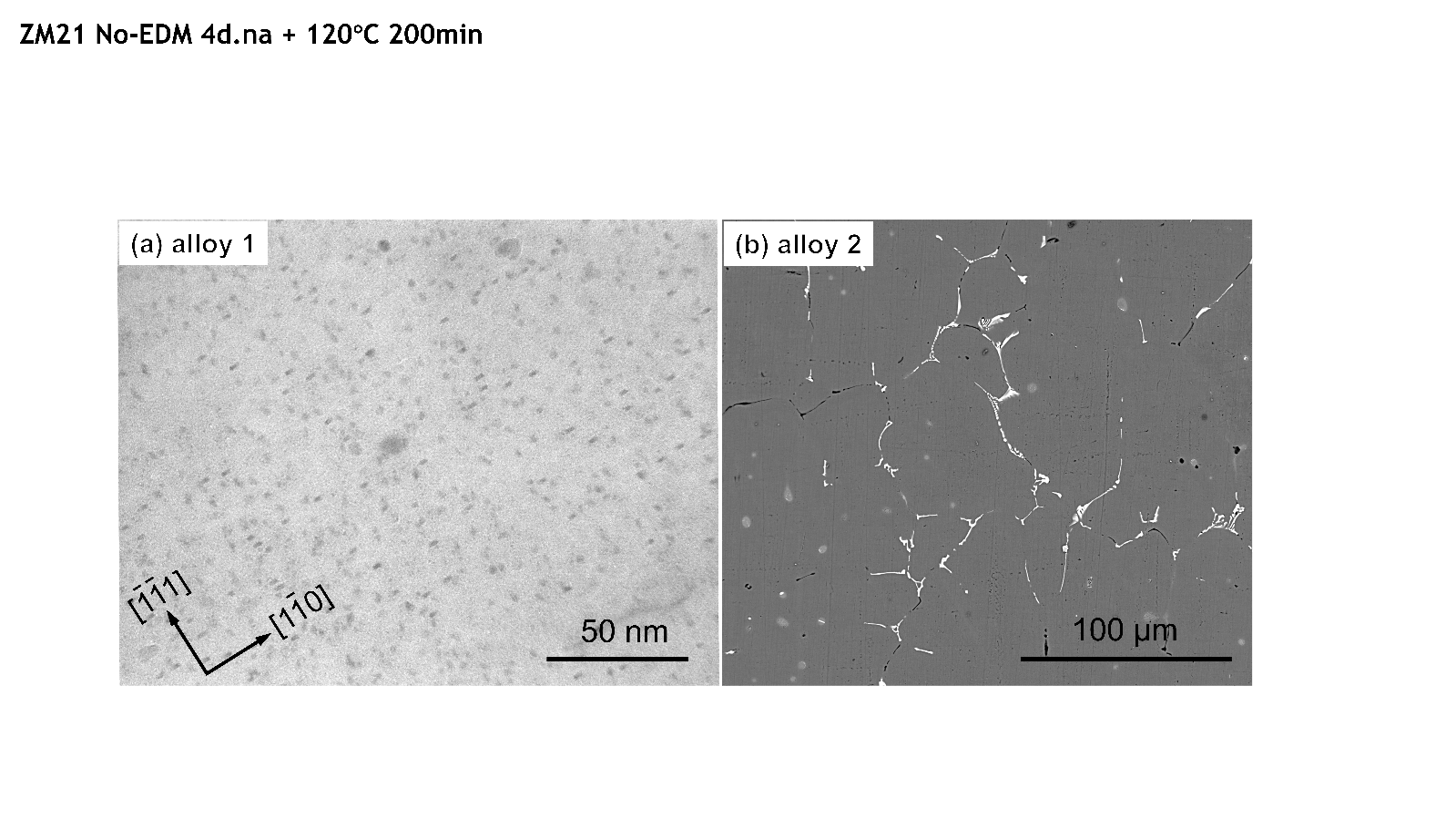


Figure 1. (a) TEM image along $\left\langle112 \right\rangle$ Al zone axis of the first alloy. (b) SEM backscattered electron image of the second alloy.

The TEM foils are prepared from two different aluminium alloy compositions. The microstructure of alloy 1, which is homogenized, quenched, and then age hardened, is shown in the Figure 1 (a) along the $\left\langle112 \right\rangle$ Al zone axis. The fine precipitates of the metastable η' phase lye with their basal plane on the $\left\{ 111 \right\}$ Al plane which is positioned edge-on with respect to the electron beam direction. The precipitates which cannot be seen as edge-on lye on the other three $\left\{ 111 \right\}$ Al planes which are inclined with respect to the electron beam. The precipitates are of plate shaped morphology with 7-8 nm diameter. There are no coarse primary or secondary intermetallic containing transition metals.

The microstructure of the alloy 2 with high content of Mn and Fe elements and other minor transition metal additions is shown in the Figure 1 (b). The backscattered electron SEM image shows the Fe-bearing intermetallic, the Mg_2_Si stable precipitate phase and the Si rich phases distributed along the grain boundaries meanwhile the finer and globular ones inside the grains.


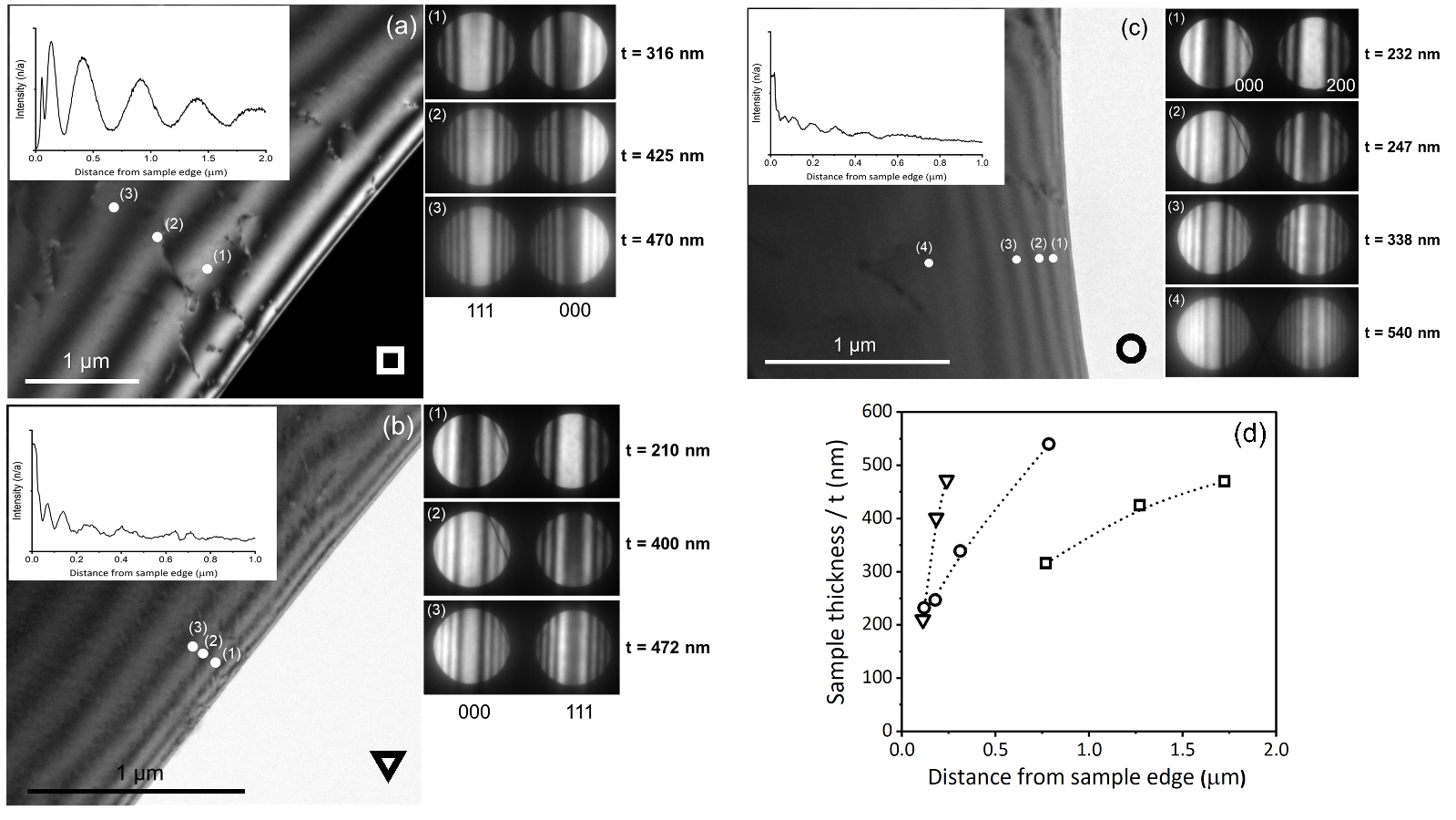


Figure 2. Thickness measurement in alloy 2. (a) Dark field image where the {111} Al spot is excited. (b) Bright field image where the {111} Al spot is excited. (c) Bright field image where the {200} Al spot is excited. The intensity fluctuation due to thickness fringes are shown in the insets of each TEM image. The two-beam CBED patterns, the calculated thickness and the sampling path are shown next to each TEM image. (d) The TEM foil thickness as a function of the distance from the sample edge for alloy 2.

Figure 2 shows the thickness measurement using two-beam condition CBED diffraction for the alloy 2. Three TEM samples were analysed. Figure 2 (a) and (b) shows the dark field and bright field image where the $\left\{ 111 \right\}$ spot is excited, meanwhile Figure 2 (c) shows the bright field image where the $\left\{ 200 \right\}$ spot is excited. The sampling path is shown in each figure where the probing points on the TEM image corresponds to the CBED diffraction points. The thickness was extracted out using electron wavelength of 0.00197 nm for 300 kV acceleration voltage and interplanar spacing of the $\left\{ 200 \right\}$ and $\left\{ 111 \right\}$ Al planes being 0.2025 nm and 0.2235 nm, respectively.

The insets in each TEM image shows the fluctuations in the intensity due to thickness fringes on the TEM images. The thickness fringes in Figure 2 (a) are widely spaced than the thickness fringes in the Figure 2 (b) and (c). Considering that the extinction distance is equal in all the cases, it indicates that the rate of increase in thickness as a function distance is smaller for sample shown in the Figure 2 (a). Therefore, the sample thickness will increase at a slower rate in Figure 2 (a) compared to Figure 2 (b) and (c). The plot shown in the Figure 2 (d) shows the sample thickness as a function of distance from the edge of the hole. The thickness of the TEM samples seems to increase abruptly to over 200 nm right next to the hole edge and then increase parabolically within 2 µm from the hole edge.
